# Changes in Glaucoma Medication during the Past Eight Years and Future Directions in Japan Based on an Insurance Medical Claim Database

**DOI:** 10.1155/2017/7642049

**Published:** 2017-10-31

**Authors:** Masako Sakamoto, Kazuyoshi Kitamura, Kenji Kashiwagi

**Affiliations:** Department of Ophthalmology, University of Yamanashi Faculty of Medicine, Chuo, Yamanashi, Japan

## Abstract

**Purpose:**

To investigate changes in the status of glaucoma care between 2006 and 2013 and to predict future directions of glaucoma care in Japan.

**Subjects and Methods:**

Japanese subjects registered in the largest national insurance claim database in Japan from 2006 to 2013 were analyzed. Estimations of the number of glaucoma patients during the past eight years and of the number of future patients were calculated. Changes in prescription trends among the same patients in the three-year period after initiating antiglaucoma medication were also investigated.

**Results:**

There was a total of 3,016,000 subjects in the database. The proportion of glaucoma patients increased consistently from 2.5% in 2006 to 4.5% in 2013. This trend was predicted to continue until 2025, followed by a constant decrease with age. The most frequently prescribed antiglaucoma medications were prostaglandin analogues (PGs); however, in recent years, fixed combination therapy has emerged as a major treatment. Among 2856 newly diagnosed glaucoma patients; 94.7% of the patients initially received a single medication, but 25% of the patients received additional medications within 3 years.

**Conclusions:**

The prevalence of glaucoma patients has significantly increased during the past eight years. The number of antiglaucoma medications continuously increased during the treatment period.

## 1. Introduction

Glaucoma is one of the major ocular diseases that results in acquired blindness. The prevalence of glaucoma among subjects 40 years of age or older is 5%, and there are estimated four million glaucoma patients in Japan [1]. Aging is one of the major risk factors for glaucoma [1, 2], and it is predicted that the prevalence of glaucoma will increase in an aging society [3].

Reduction of intraocular pressure is the only proven treatment for glaucoma [4]. Glaucoma treatments consist of ophthalmic solutions, laser treatments, and surgical procedures. We have described the changes in antiglaucoma ophthalmic solutions prescribed in Japan in 2010 [5]. The glaucoma treatment regimen has markedly improved, as exemplified by new antiglaucoma ophthalmic solutions and minimally invasive glaucoma surgeries becoming available during the past years [6, 7]. Unfortunately, few studies have investigated the current status of glaucoma care, including changes in the number of glaucoma patients and large-scale treatment regimens in Japan.

It is important to investigate changes in glaucoma treatment from medical and surgical viewpoints to elucidate the current tasks of glaucoma care and to assist with future predictions of the glaucoma burden; we accomplished this aim by analyzing national insurance claim data in the present study.

## 2. Subjects and Methods

This study adhered to the tenets of the Declaration of Helsinki and was approved by the University of Yamanashi Ethical Review Board. Because the data used in this study do not contain any personal information, the Ethical Review Board agreed to permit this study without requiring written informed consent from all patients.

### 2.1. Database

In this study, we used the claim database of the Japan Medical Data Center (JMDC), established in 2003, to accumulate receipt data and perform epidemiological and health service research [8]. The JMDC is the largest medical database in Japan, and the details of this database have been described elsewhere [5, 8, 9]. In brief, the database employs an anonymous linkage system using an encryption code and combines individual medical claim information from different health insurance societies through a computer-aided, post-entry standardization method. This database covers employees and their dependents who belong to Japan Health Insurance Society, one of two major Health Insurance systems in Japan. The data include the patients' encrypted personal identifiers, age, sex, and International Classification of Diseases version 10 (ICD-10) diagnosis codes, the names of dispensed drugs, and the size of medical institutions. The encrypted personal identifiers could be used to link claim data from different hospitals, clinics, and pharmacies.

### 2.2. Subjects

The total number of subjects registered in the database from January 2006 to December 2013 was 3,016,666.

### 2.3. Definition of Glaucoma

Glaucoma was defined as having any of glaucoma diagnosis according to the ICD-10 diagnosis codes (Supplemental Table 1 available online at https://doi.org/10.1155/2017/7642049), having a history of antiglaucoma medication use and/or glaucoma surgery (including laser treatment) and having a three-month or longer history of any glaucoma treatment, including an antiglaucoma prescription, laser procedures, and glaucoma surgeries. Patients who met the following criteria were excluded from the analysis: patients who changed insurance systems, those who had no records in the Japan Health Insurance Society database, and those whose glaucoma diagnosis were retracted. The details of data extraction have been shown elsewhere [8, 9].

### 2.4. Data Adjustment

The estimated number of glaucoma patients in each study year was adjusted based on the 2010 Japanese census to compare proportions among years during the study period. Future trends in the estimated number of glaucoma patients were evaluated based on a combination of data from the 2013 database and official prediction data from the National Institute of Population and Social Security Research based on birth and mortality rates in January 2012.

### 2.5. Changes in Antiglaucoma Ophthalmic Solutions during a Three-Year Period among Newly Diagnosed and Prescribed Glaucoma Patients

Subjects with newly diagnosed glaucoma were defined as having belonged to the Japan Health Insurance Society union for more than one year before entry, having no history of any type of glaucoma, having been prescribed antiglaucoma ophthalmic solutions, and having no laser and/or surgical treatments recorded in the JMDC database. Among glaucoma patients who were newly diagnosed, those who had been registered in the database were evaluated to determine changes made to their medical treatment regimen during a three-year period. Glaucoma subjects with a history of any laser or surgical treatments over the three-year period were excluded from this study because of investigating changing in medication precisely.

### 2.6. Statistical Analysis

For the statistical analysis, all commercially available ophthalmic solutions in Japan were classified into the following categories: prostaglandin analogues (PGs), beta-blocker (BB), carbonic anhydrase inhibitor (CAI), and others (Supplemental Table 2).

The data were analyzed with JMP 11.0 software (SAS Institute Inc., Cary, NC, USA), and values are presented as the means ± standard deviations. Correlations between the proportion of glaucoma patients, frequency of glaucoma medication, and glaucoma surgery and the changes in the number of antiglaucoma ophthalmic solutions per patient were evaluated using Pearson's correlation coefficient or the Wilcoxon signed-rank test. *P* values less than 0.05 were considered statistically significant.

## 3. Results

### 3.1. Changes in the Estimated Number of Glaucoma Patients

**Figure 1:**
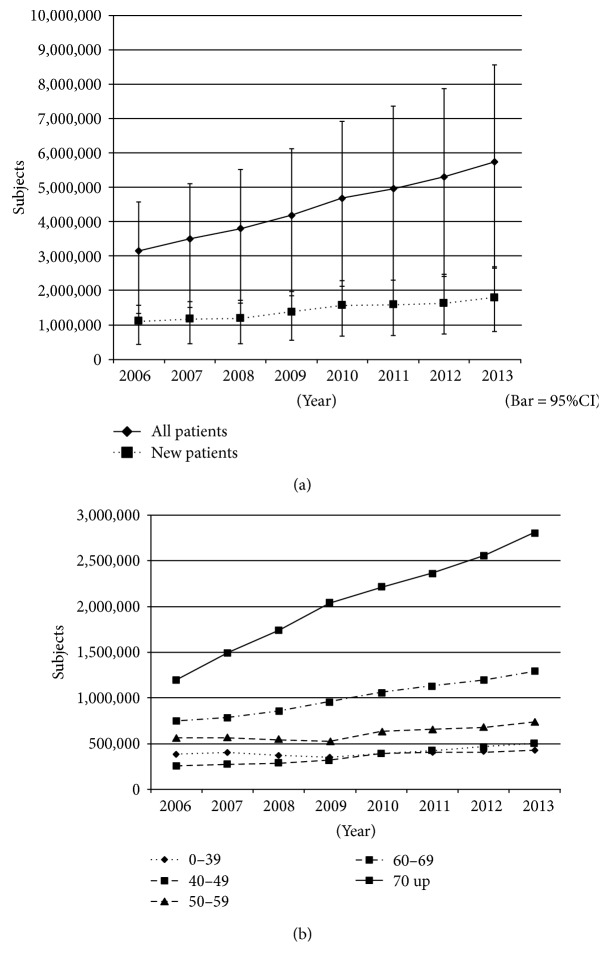
Changes in the estimated number of glaucoma patients. (a) Changes in the estimated number of all glaucoma patients and of newly diagnosed glaucoma patients. (b) Changes in the estimated number of glaucoma patients among generations. Bar = SD.

The estimated number of glaucoma patients in 2006 was 3,140,000. This number significantly (*P* < 0.0001) increased by year, reaching 5,750,000 in 2013 (Figure 1a). When comparing age groups, the estimated number of glaucoma patients did not significantly change among patients younger than 50 years old, but the number of patients the same or older than 50 years of age significantly increased each year over the 7-year period between 2006 and 2016 (*P* < 0.0001); the magnitude of the increase was greater with age (Figure 1(b)).

### 3.2. Future Prediction of the Estimated Number of Glaucoma Patients

**Figure 2:**
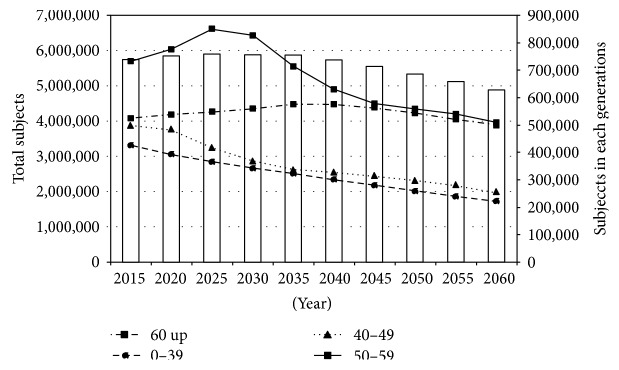
Future prediction of the number of glaucoma patients.

The number of glaucoma patients was predicted to increase to approximately 5,890,000 by 2025, and thereafter, it was predicted to decrease due to a reduction of the population. Changes in profiles were not the same among age groups. The number of patients aged 60 or older was expected to increase by 2035, whereas the profile of those under 50 years of age showed a consistent reduction during the study period (Figure 2).

### 3.3. Comparison of Ratios of Medicated Patients among Age Groups

The ratio of glaucoma patients using any medications significantly increased with age (*P* < 0.001). The rates of glaucoma patients using any medications among less than 40 years old, 40–49 years old, 50–59 years old, 60–69 years old, and 70 years old or older were 19.3%, 37.1%, 48.1%, 53.4%, and 58.0%, respectively.

### 3.4. Changes in the Number of Prescribed Antiglaucoma Ophthalmic Solutions per Patient

The number of antiglaucoma agents significantly increased during the study period. The introduction of fixed combination therapy drastically changed the trend in the prescription of antiglaucoma ophthalmic solutions. The number of prescribed ophthalmic solutions per patient was 1.49 agents in 2006, which was relatively constant by 2009; however, after 2010, when fixed combination ophthalmic solutions became available in Japan, the number of antiglaucoma agents was increased, reaching 1.71 in 2013.

### 3.5. Trends in Antiglaucoma Ophthalmic Solutions

**Figure 3:**
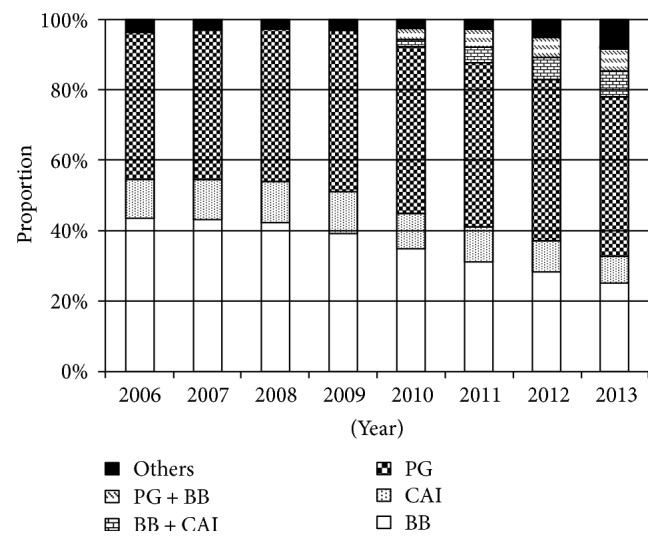
Trends in antiglaucoma ophthalmic solutions. PG: prostaglandin analogue; BB: beta blocker; CAI: carbonic anhydrase inhibitor.

Figure 3 shows the yearly change in the contents of antiglaucoma ophthalmic solutions. PGs, BBs, and CAIs were the major antiglaucoma ophthalmic solutions used throughout the study period. The proportion of PGs was similar, while those of BBs and CAIs gradually decreased during the study period. The proportion of fixed combination therapy significantly increased after its introduction in 2010. An analysis based on antiglaucoma agents showed that PGs and PG-containing fixed combination therapy accounted for 51.8% of all antiglaucoma agents in 2013.

### 3.6. Frequency of Glaucoma Surgery

Because the number of glaucoma surgeries was limited for each year, the total number of glaucoma surgeries during the study period was included in the analysis. The mean frequency of glaucoma surgery was 0.57%, but the frequency of glaucoma surgery significantly increased with age, with a peak at 0.70% in patients in their 60s.

### 3.7. Changes in Antiglaucoma Ophthalmic Solutions during a Three-Year Period among Newly Diagnosed and Prescribed Glaucoma Patients

**Figure 4:**
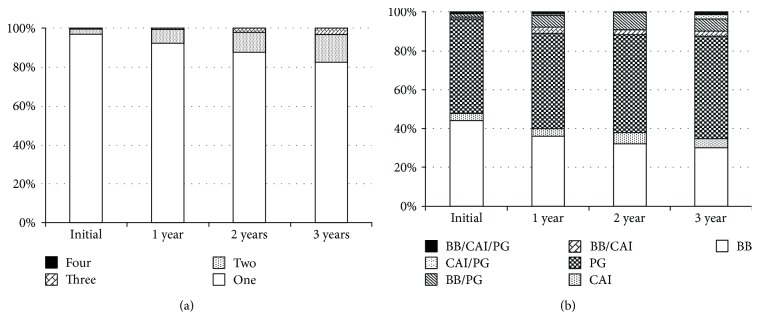
Change in antiglaucoma ophthalmic solutions in three years among newly medicated patients. (a) Change in bottles of antiglaucoma ophthalmic solution. (b) Change in the number of antiglaucoma ophthalmic agents. PG: prostaglandin analogue; BB: beta blocker; CAI: carbonic anhydrase inhibitor.

A total of 2856 glaucoma patients were eligible for the analysis. Almost all newly medicated glaucoma patients (96.6%) received a single antiglaucoma ophthalmic solution. In the three-year follow up, 14.1% of these patients were administered the same or more than two antiglaucoma ophthalmic solutions (Figure 4(a)). There were 1.03 ± 0.20 bottles of antiglaucoma ophthalmic solutions and 1.06 ± 0.26 types of agents during the first year. By contrast, after three years of follow-up, the numbers were 1.21 ± 0.48 bottles and 1.35 ± 0.68 types, respectively. The numbers of antiglaucoma ophthalmic solutions and agents significantly increased during the first three years (*P* < 0.0001, Wilcoxon signed-rank test). PGs were the most popular type of antiglaucoma ophthalmic solution, followed by BBs, but the treatment regimens became complicated during the three-year period (Figure 4(b)).

## 4. Discussion

The current study revealed the number of Japanese glaucoma patients in recent years and the frequencies of medications, and surgeries among these patients. The proportion of glaucoma patients significantly increased during the eight years of the study, especially among elderly subjects. The total number of patients of glaucoma patients increased with age. It is expected to increase until 2025 and decrease thereafter due to the aging and reduction of birthrate of Japanese society.

In recent years, many antiglaucoma ophthalmic solutions have become available, resulting in diversification of antiglaucoma ophthalmic solutions. The frequency of glaucoma medication significantly increased with age, and the total number of antiglaucoma agents increased. Therefore, medical expenses for glaucoma treatment will likely increase, and the development of less expensive and more effective therapies for glaucoma is needed.

The frequency of glaucoma surgery increased with age as expected, but the frequency of glaucoma surgery did not significantly change during the study period. Several new surgeries had been introduced recently, which may drastically change glaucoma care. Therefore, it is important to investigate changes in glaucoma surgery. However, the number of glaucoma surgery was not sufficient to investigate this in the present study. Further investigations should be necessary. The introduction of new antiglaucoma ophthalmic solutions may have contributed to this finding. New surgical approaches have been developed recently, including minimally invasive glaucoma surgery, which has been reported to be effective for IOP reduction without severe complications [6, 10, 11]. Although new glaucoma surgeries have not been widely applied in Japan, this trend may change glaucoma care in the near future.

The current study revealed that PGs occupied a major position among antiglaucoma ophthalmic solutions. BBs and CAIs were second and third, respectively, at the beginning of the study period, but their use was gradually reduced over time.

Recently, new categories of antiglaucoma ophthalmic solutions have become available, such as an alpha 2 agonist and fixed combinations. It has been reported that the alpha 2 agonist brimonidine exerts an IOP-independent neuroprotective effect [12]. It is well known that normal tension glaucoma is the most common glaucoma subtype, especially in Asian countries, including Japan [1, 13-17]. Antiglaucoma medications with an IOP-independent neuroprotective effect are highly preferred for glaucoma treatment. The ROCK inhibitor, which is an ophthalmic solution that reduces the IOP by enhancing conventional outflow, became available in Japan very recently and has been reported to show an additional IOP reduction in combination with other antiglaucoma ophthalmic solutions [18-21]. Thus, the prescription trend for antiglaucoma ophthalmic solutions may change in the future.

The number of antiglaucoma agents of newly-medicated glaucoma patients increased significantly during a three-year period; 20.4% of glaucoma patients treated by one antiglaucoma agent required two or more agents during this period. It has been noted that increasing the number of ophthalmic solutions results in deterioration of adherence and/or continuity, as well as increasing the risk of side effects [22, 23]. It will be important to develop better treatments that control the IOP using fewer antiglaucoma ophthalmic solutions.

The results of this study demonstrate that the number of glaucoma patients increased during the study period, which also indicates that the medical expenses may accelerate in the future. Comprehensive new glaucoma treatments involving medical, surgical, and laser approaches should be investigated from a socioeconomic perspective.

The results of this study cannot be directly compared to data from epidemiological studies because the current study employed claim data. The accuracy of diagnosis in the current study may be lower than that of an epidemiological study. We could not obtain some important data from the currently employed database including severity of glaucomatous optic neuropathy, visual filed defect, visual acuity, IOP, and other glaucoma-related parameters, which could result in some limitations in the current study. The claim data in the current study were limited to patients in the Social Health Insurance Database. Therefore, we cannot eliminate some possibility of bias in terms of both subject selection and future prediction completely. All Japanese citizens are obligated to register for public insurance systems. Two main types of public insurance systems are available in Japan: National Health Insurance for retired persons and the self-employed and their dependents and Employee Health Insurance for employees and their dependents. It is difficult to collect combined data from subjects registered in the National Insurance System because data are managed independently by local autonomies. No clear evidence is available that demonstrates the differences between the two insurance systems regarding subject characteristics, including socioeconomic status and disease prevalence. Therefore, the status of glaucoma care between two insurance systems could be similar. Ratios of medicated or operated patients seem to be fewer than expected. Possible explanations could be considered. Firstly, prevalence of patients with normal tension glaucoma (NTG) is higher in Japan compared to other countries [1]. The rate of progress of glaucomatous optic neuropathy among patients with NTG is generally slower than that among those with other types of glaucoma. Secondly, advancement of apparatus such as optical coherence tomography may result in much frequent discovery of very early stage of glaucoma named preperimetric glaucoma which is sometimes subject to observation Thirdly, there were some patients who had been simply followed up after well-performed glaucoma surgery. It is impossible to judge if patients had a history of glaucoma surgery before 2006. Lastly, physicians may not terminate glaucoma diagnosis due to some reasons.

## 5. Conclusion

This study revealed the current status of glaucoma care in Japan. The proportion of glaucoma patients has significantly increased during the past eight years, but it may begin to decrease after 2025. PGs were the most frequently prescribed antiglaucoma medications, and the number of antiglaucoma medications continuously increased during the treatment period. These data may be adapted to other countries. It is necessary to pursue a more effective and less expensive glaucoma care system by developing new treatment methods and a better screening system.

## Supplementary Material

Supplemental Table 1. Supplemental Table 2.
