# Supplementary material for: Changes in Glaucoma Medication during the Past Eight Years and Future Directions in Japan Based on an Insurance Medical Claim Database
Source: J Ophthalmol. 2017 Oct 31;2017:7642049. doi: 10.1155/2017/7642049 (PMC5684567; doi:10.1155/2017/7642049)
Supplement: Supplementary file 1 — Supplemental Table 1. Supplemental Table 2. [file 7642049.f1.pdf]

Supplemental Tables

Supplemental Table 1

Classification #	Definition
H40.0	Glaucoma suspect
H40.1	Primary open-angle glaucoma
H40.3	Glaucoma secondary to eye trauma
H40.4	Glaucoma secondary to eye inflammation
H40.5	Glaucoma secondary to other eye disorders
H40.6	Glaucoma secondary to drugs
H40.8	Other glaucoma
H40.9	Glaucoma, unspecified
H42	Glaucoma in diseases classified elsewhere
H42.0	Glaucoma in endocrine, nutritional and metabolic diseases
H42.8	Glaucoma in other diseases classified elsewhere

Supplemental Table 2

Name of compound	Category
Isopropyl unoprostoneone	PG
Nipradilol	BB
Brinzolamide	CAI
Timolol maleate	BB
Latanoprost	PG
Apraclonidine hydrochloride	others
Carteolol hydrochloride	BB
Dipivefrin Hydrochloride	others
Dorzolamide hydrochloride	CAI
Pilocarpine hydrochloride	others
Bunazosin hydrochloride	BB
Levobunolol hydrochloride	BB
Distigmine bromide	Others
Travoprost	PG
Tafluprost	PG
Bimatoprost	PG
Timolol maleate/latanoprost	PG + BB
Timolol maleate/dorzolamide hydrochloride	BB + CAI
Timolol maleate//travoprost	PG + BB
